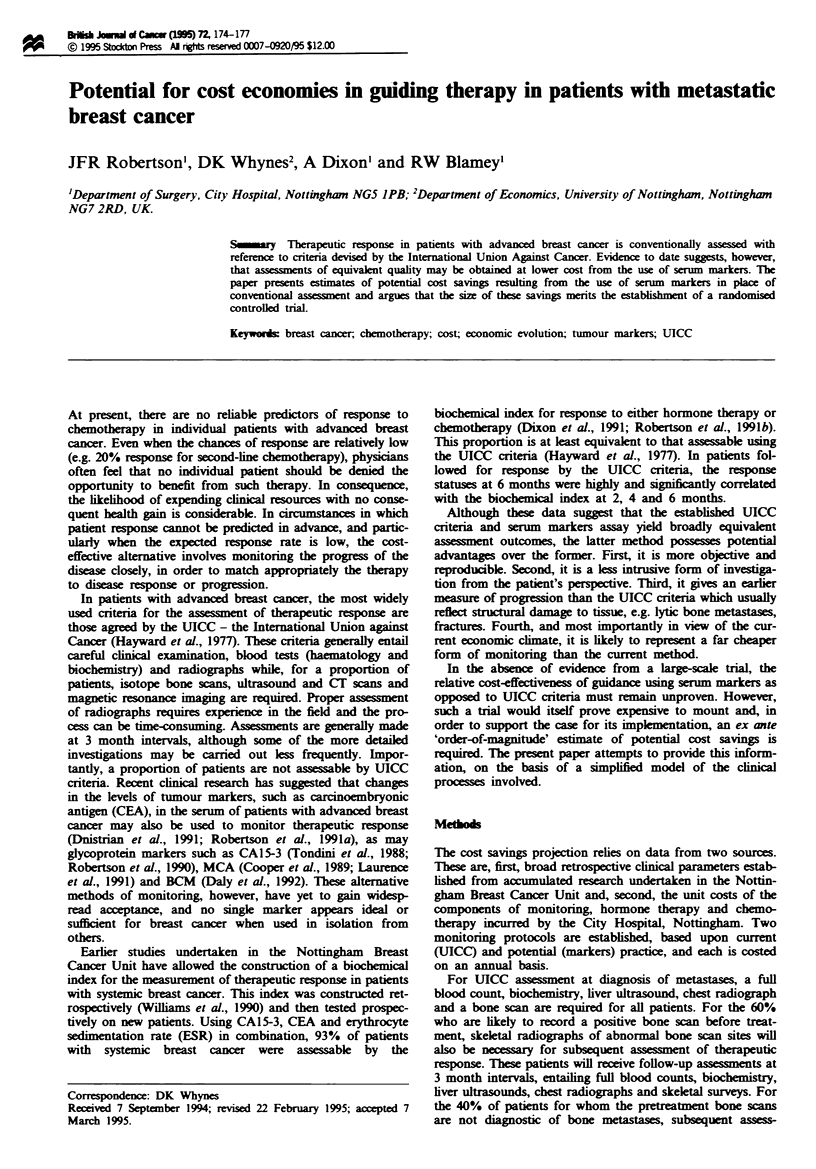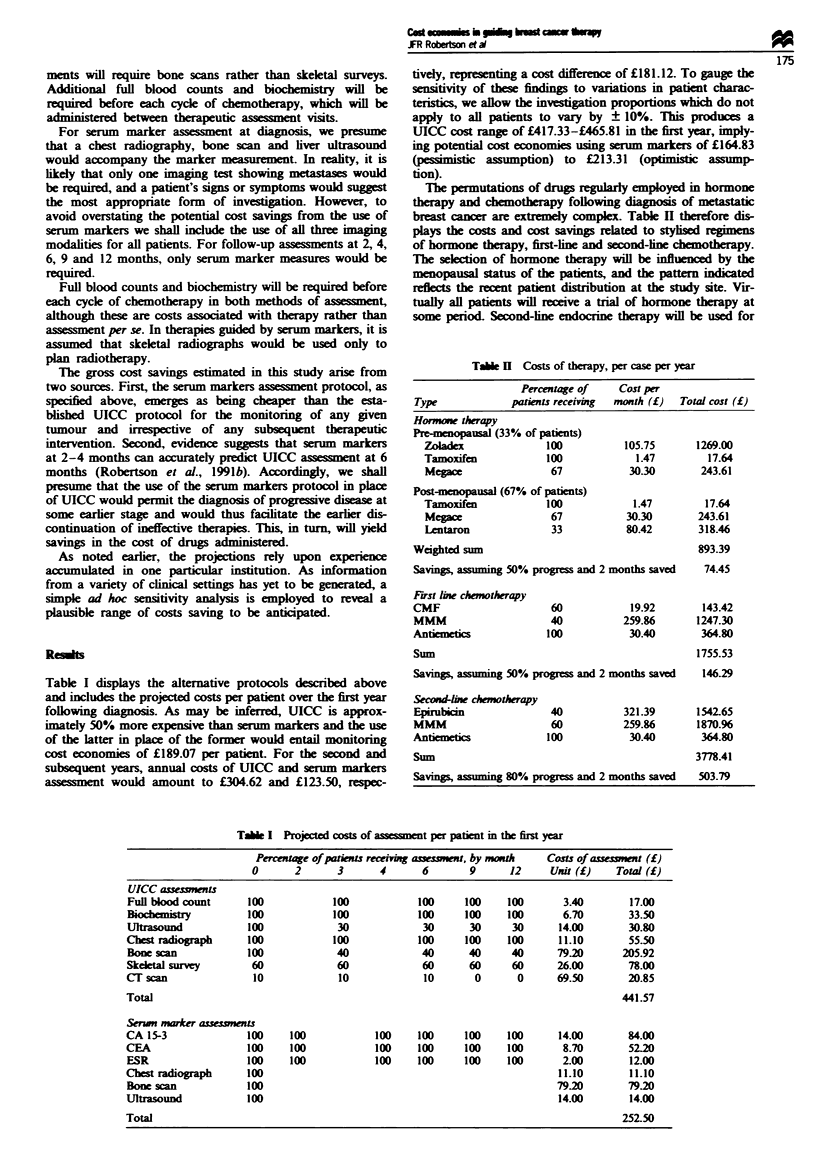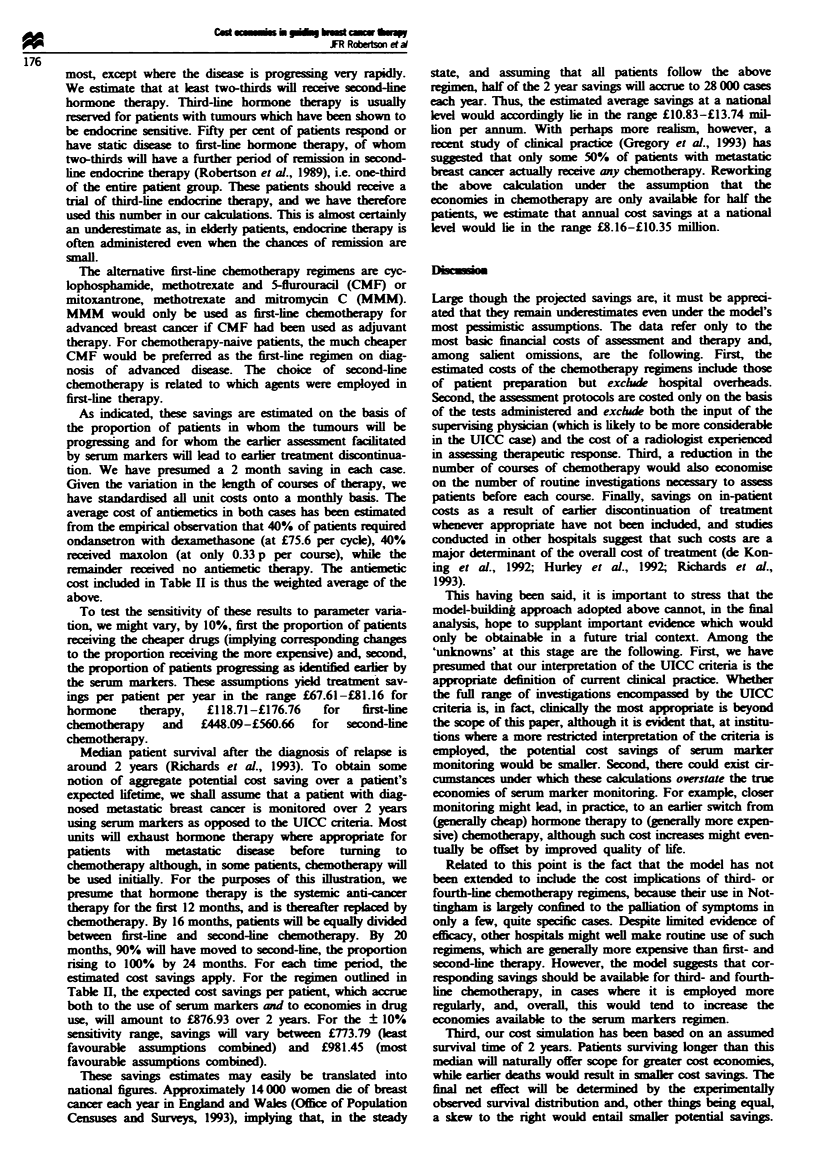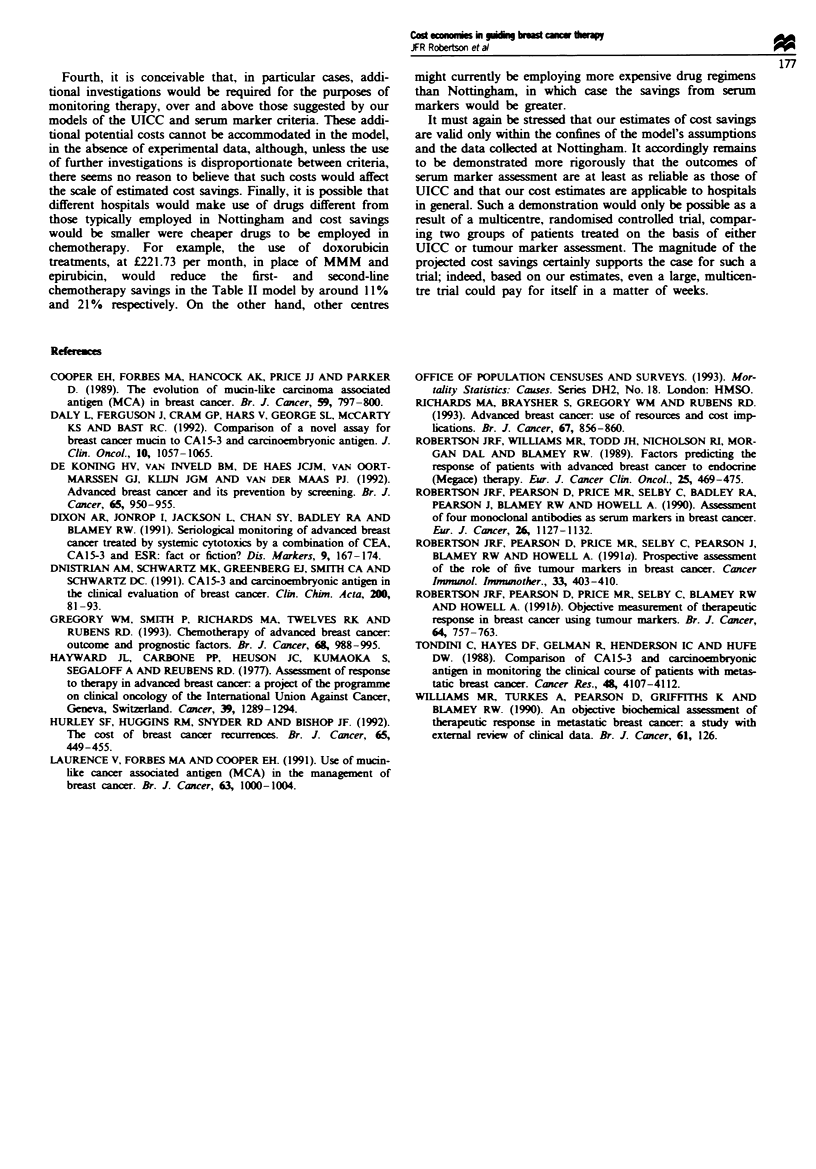# Potential for cost economies in guiding therapy in patients with metastatic breast cancer.

**DOI:** 10.1038/bjc.1995.297

**Published:** 1995-07

**Authors:** J. F. Robertson, D. K. Whynes, A. Dixon, R. W. Blamey

**Affiliations:** Department of Surgery, City Hospital, Nottingham, UK.

## Abstract

Therapeutic response in patients with advanced breast cancer is conventionally assessed with reference to criteria devised by the International Union Against Cancer. Evidence to date suggests, however, that assessments of equivalent quality may be obtained at lower cost from the use of serum markers. The paper presents estimates of potential cost savings resulting from the use of serum markers in place of conventional assessment and argues that the size of these savings merits the establishment of a randomised controlled trial.


© 1995 Stockton Press All rights reserved 0007–0920/95 $12.00

### Potential for cost economies in guiding therapy in patients with metastatic breast cancer

JFR Robertson[1], DK Whynes[2], A Dixon[1] and RW Blamey[1]

[1]Department of Surgery, City Hospital, Nottingham NG5 1PB; [2]Department of Economics, University of Nottingham, Nottingham NG7 2RD, UK.

**Summary** Therapeutic response in patients with advanced breast cancer is conventionally assessed with reference to criteria devised by the International Union Against Cancer. Evidence to date suggests, however, that assessments of equivalent quality may be obtained at lower cost from the use of serum markers. The paper presents estimates of potential cost savings resulting from the use of serum markers in place of conventional assessment and argues that the size of these savings merits the establishment of a randomised controlled trial.

**Keywords:** breast cancer; chemotherapy; cost; economic evolution; tumour markers; UICC

At present, there are no reliable predictors of response to chemotherapy in individual patients with advanced breast cancer. Even when the chances of response are relatively low (e.g. 20% response for second-line chemotherapy), physicians often feel that no individual patient should be denied the opportunity to benefit from such therapy. In consequence, the likelihood of expending clinical resources with no consequent health gain is considerable. In circumstances in which patient response cannot be predicted in advance, and particularly when the expected response rate is low, the cost-effective alternative involves monitoring the progress of the disease closely, in order to match appropriately the therapy to disease response or progression.

In patients with advanced breast cancer, the most widely used criteria for the assessment of therapeutic response are those agreed by the UICC – the International Union against Cancer (Hayward *et al*., 1977). These criteria generally entail careful clinical examination, blood tests (haematology and biochemistry) and radiographs while, for a proportion of patients, isotope bone scans, ultrasound and CT scans and magnetic resonance imaging are required. Proper assessment of radiographs requires experience in the field and the process can be time-consuming. Assessments are generally made at 3 month intervals, although some of the more detailed investigations may be carried out less frequently. Importantly, a proportion of patients are not assessable by UICC criteria. Recent clinical research has suggested that changes in the levels of tumour markers, such as carcinoembryonic antigen (CEA), in the serum of patients with advanced breast cancer may also be used to monitor therapeutic response (Dnistrian *et al*., 1991; Robertson *et al*., 1991*a*), as may glycoprotein markers such as CA15-3 (Tondini *et al*., 1988; Robertson *et al*., 1990), MCA (Cooper *et al*., 1989; Laurence *et al*., 1991) and BCM (Daly *et al*., 1992). These alternative methods of monitoring, however, have yet to gain widespread acceptance, and no single marker appears ideal or sufficient for breast cancer when used in isolation from others.

Earlier studies undertaken in the Nottingham Breast Cancer Unit have allowed the construction of a biochemical index for the measurement of therapeutic response in patients with systemic breast cancer. This index was constructed retrospectively (Williams *et al*., 1990) and then tested prospectively on new patients. Using CA15-3, CEA and erythrocyte sedimentation rate (ESR) in combination, 93% of patients with systemic breast cancer were assessable by the biochemical index for response to either hormone therapy or chemotherapy (Dixon *et al*., 1991; Robertson *et al*., 1991*b*). This proportion is at least equivalent to that assessable using the UICC criteria (Hayward *et al*., 1977). In patients followed for response by the UICC criteria, the response statuses at 6 months were highly and significantly correlated with the biochemical index at 2, 4 and 6 months.

Although these data suggest that the established UICC criteria and serum markers assay yield broadly equivalent assessment outcomes, the latter method possesses potential advantages over the former. First, it is more objective and reproducible. Second, it is a less intrusive form of investigation from the patient's perspective. Third, it gives an earlier measure of progression than the UICC criteria which usually reflect structural damage to tissue, e.g. lytic bone metastases, fractures. Fourth, and most importantly in view of the current economic climate, it is likely to represent a far cheaper form of monitoring than the current method.

In the absence of evidence from a large-scale trial, the relative cost-effectiveness of guidance using serum markers as opposed to UICC criteria must remain unproven. However, such a trial would itself prove expensive to mount and, in order to support the case for its implementation, an *ex ante* 'order-of-magnitude' estimate of potential cost savings is required. The present paper attempts to provide this information, on the basis of a simplified model of the clinical processes involved.

### Methods

The cost savings projection relies on data from two sources. These are, first, broad retrospective clinical parameters established from accumulated research undertaken in the Nottingham Breast Cancer Unit and, second, the unit costs of the components of monitoring, hormone therapy and chemotherapy incurred by the City Hospital, Nottingham. Two monitoring protocols are established, based upon current (UICC) and potential (markers) practice, and each is costed on an annual basis.

For UICC assessment at diagnosis of metastases, a full blood count, biochemistry, liver ultrasound, chest radiograph and a bone scan are required for all patients. For the 60% who are likely to record a positive bone scan before treatment, skeletal radiographs of abnormal bone scan sites will also be necessary for subsequent assessment of therapeutic response. These patients will receive follow-up assessments at 3 month intervals, entailing full blood counts, biochemistry, liver ultrasounds, chest radiographs and skeletal surveys. For the 40% of patients for whom the pretreatment bone scans are not diagnostic of bone metastases, subsequent assess-

Correspondence: DK Whynes
Received 7 September 1994; revised 22 February 1995; accepted 7 March 1995.

ments will require bone scans rather than skeletal surveys. Additional full blood counts and biochemistry will be required before each cycle of chemotherapy, which will be administered between therapeutic assessment visits.

For serum marker assessment at diagnosis, we presume that a chest radiography, bone scan and liver ultrasound would accompany the marker measurement. In reality, it is likely that only one imaging test showing metastases would be required, and a patient's signs or symptoms would suggest the most appropriate form of investigation. However, to avoid overstating the potential cost savings from the use of serum markers we shall include the use of all three imaging modalities for all patients. For follow-up assessments at 2, 4, 6, 9 and 12 months, only serum marker measures would be required.

Full blood counts and biochemistry will be required before each cycle of chemotherapy in both methods of assessment, although these are costs associated with therapy rather than assessment *per se*. In therapies guided by serum markers, it is assumed that skeletal radiographs would be used only to plan radiotherapy.

The gross cost savings estimated in this study arise from two sources. First, the serum markers assessment protocol, as specified above, emerges as being cheaper than the established UICC protocol for the monitoring of any given tumour and irrespective of any subsequent therapeutic intervention. Second, evidence suggests that serum markers at 2–4 months can accurately predict UICC assessment at 6 months (Robertson *et al.*, 1991*b*). Accordingly, we shall presume that the use of the serum markers protocol in place of UICC would permit the diagnosis of progressive disease at some earlier stage and would thus facilitate the earlier discontinuation of ineffective therapies. This, in turn, will yield savings in the cost of drugs administered.

As noted earlier, the projections rely upon experience accumulated in one particular institution. As information from a variety of clinical settings has yet to be generated, a simple *ad hoc* sensitivity analysis is employed to reveal a plausible range of costs saving to be anticipated.

### Results

Table I displays the alternative protocols described above and includes the projected costs per patient over the first year following diagnosis. As may be inferred, UICC is approximately 50% more expensive than serum markers and the use of the latter in place of the former would entail monitoring cost economies of £189.07 per patient. For the second and subsequent years, annual costs of UICC and serum markers assessment would amount to £304.62 and £123.50, respectively, representing a cost difference of £181.12. To gauge the sensitivity of these findings to variations in patient characteristics, we allow the investigation proportions which do not apply to all patients to vary by ± 10%. This produces a UICC cost range of £417.33–£465.81 in the first year, implying potential cost economies using serum markers of £164.83 (pessimistic assumption) to £213.31 (optimistic assumption).

The permutations of drugs regularly employed in hormone therapy and chemotherapy following diagnosis of metastatic breast cancer are extremely complex. Table II therefore displays the costs and cost savings related to stylised regimens of hormone therapy, first-line and second-line chemotherapy. The selection of hormone therapy will be influenced by the menopausal status of the patients, and the pattern indicated reflects the recent patient distribution at the study site. Virtually all patients will receive a trial of hormone therapy at some period. Second-line endocrine therapy will be used for

**Table II** Costs of therapy, per case per year

| *Type* | *Percentage of patients receiving* | *Cost per month (£)* | *Total cost (£)* |
|---|---|---|---|
| *Hormone therapy* | | | |
| Pre-menopausal (33% of patients) | | | |
| Zoladex | 100 | 105.75 | 1269.00 |
| Tamoxifen | 100 | 1.47 | 17.64 |
| Megace | 67 | 30.30 | 243.61 |
| Post-menopausal (67% of patients) | | | |
| Tamoxifen | 100 | 1.47 | 17.64 |
| Megace | 67 | 30.30 | 243.61 |
| Lentaron | 33 | 80.42 | 318.46 |
| Weighted sum | | | 893.39 |
| Savings, assuming 50% progress and 2 months saved | | | 74.45 |
| *First line chemotherapy* | | | |
| CMF | 60 | 19.92 | 143.42 |
| MMM | 40 | 259.86 | 1247.30 |
| Antiemetics | 100 | 30.40 | 364.80 |
| Sum | | | 1755.53 |
| Savings, assuming 50% progress and 2 months saved | | | 146.29 |
| *Second-line chemotherapy* | | | |
| Epirubicin | 40 | 321.39 | 1542.65 |
| MMM | 60 | 259.86 | 1870.96 |
| Antiemetics | 100 | 30.40 | 364.80 |
| Sum | | | 3778.41 |
| Savings, assuming 80% progress and 2 months saved | | | 503.79 |

**Table I** Projected costs of assessment per patient in the first year

| | *Percentage of patients receiving assessment, by month* | | | | | | | *Costs of assessment (£)* | |
|---|---|---|---|---|---|---|---|---|---|
| | *0* | *2* | *3* | *4* | *6* | *9* | *12* | *Unit (£)* | *Total (£)* |
| *UICC assessments* | | | | | | | | | |
| Full blood count | 100 | | 100 | | 100 | 100 | 100 | 3.40 | 17.00 |
| Biochemistry | 100 | | 100 | | 100 | 100 | 100 | 6.70 | 33.50 |
| Ultrasound | 100 | | 30 | | 30 | 30 | 30 | 14.00 | 30.80 |
| Chest radiograph | 100 | | 100 | | 100 | 100 | 100 | 11.10 | 55.50 |
| Bone scan | 100 | | 40 | | 40 | 40 | 40 | 79.20 | 205.92 |
| Skeletal survey | 60 | | 60 | | 60 | 60 | 60 | 26.00 | 78.00 |
| CT scan | 10 | | 10 | | 10 | 0 | 0 | 69.50 | 20.85 |
| Total | | | | | | | | | 441.57 |
| *Serum marker assessments* | | | | | | | | | |
| CA 15-3 | 100 | 100 | | 100 | 100 | 100 | 100 | 14.00 | 84.00 |
| CEA | 100 | 100 | | 100 | 100 | 100 | 100 | 8.70 | 52.20 |
| ESR | 100 | 100 | | 100 | 100 | 100 | 100 | 2.00 | 12.00 |
| Chest radiograph | 100 | | | | | | | 11.10 | 11.10 |
| Bone scan | 100 | | | | | | | 79.20 | 79.20 |
| Ultrasound | 100 | | | | | | | 14.00 | 14.00 |
| Total | | | | | | | | | 252.50 |

most, except where the disease is progressing very rapidly. We estimate that at least two-thirds will receive second-line hormone therapy. Third-line hormone therapy is usually reserved for patients with tumours which have been shown to be endocrine sensitive. Fifty per cent of patients respond or have static disease to first-line hormone therapy, of whom two-thirds will have a further period of remission in second-line endocrine therapy (Robertson *et al.*, 1989), i.e. one-third of the entire patient group. These patients should receive a trial of third-line endocrine therapy, and we have therefore used this number in our calculations. This is almost certainly an underestimate as, in elderly patients, endocrine therapy is often administered even when the chances of remission are small.

The alternative first-line chemotherapy regimens are cyclophosphamide, methotrexate and 5-flurouracil (CMF) or mitoxantrone, methotrexate and mitromycin C (MMM). MMM would only be used as first-line chemotherapy for advanced breast cancer if CMF had been used as adjuvant therapy. For chemotherapy-naive patients, the much cheaper CMF would be preferred as the first-line regimen on diagnosis of advanced disease. The choice of second-line chemotherapy is related to which agents were employed in first-line therapy.

As indicated, these savings are estimated on the basis of the proportion of patients in whom the tumours will be progressing and for whom the earlier assessment facilitated by serum markers will lead to earlier treatment discontinuation. We have presumed a 2 month saving in each case. Given the variation in the length of courses of therapy, we have standardised all unit costs onto a monthly basis. The average cost of antiemetics in both cases has been estimated from the empirical observation that 40% of patients required ondansetron with dexamethasone (at £75.6 per cycle), 40% received maxolon (at only 0.33 p per course), while the remainder received no antiemetic therapy. The antiemetic cost included in Table II is thus the weighted average of the above.

To test the sensitivity of these results to parameter variation, we might vary, by 10%, first the proportion of patients receiving the cheaper drugs (implying corresponding changes to the proportion receiving the more expensive) and, second, the proportion of patients progressing as identified earlier by the serum markers. These assumptions yield treatment savings per patient per year in the range £67.61–£81.16 for hormone therapy, £118.71–£176.76 for first-line chemotherapy and £448.09–£560.66 for second-line chemotherapy.

Median patient survival after the diagnosis of relapse is around 2 years (Richards *et al.*, 1993). To obtain some notion of aggregate potential cost saving over a patient's expected lifetime, we shall assume that a patient with diagnosed metastatic breast cancer is monitored over 2 years using serum markers as opposed to the UICC criteria. Most units will exhaust hormone therapy where appropriate for patients with metastatic disease before turning to chemotherapy although, in some patients, chemotherapy will be used initially. For the purposes of this illustration, we presume that hormone therapy is the systemic anti-cancer therapy for the first 12 months, and is thereafter replaced by chemotherapy. By 16 months, patients will be equally divided between first-line and second-line chemotherapy. By 20 months, 90% will have moved to second-line, the proportion rising to 100% by 24 months. For each time period, the estimated cost savings apply. For the regimen outlined in Table II, the expected cost savings per patient, which accrue both to the use of serum markers *and* to economies in drug use, will amount to £876.93 over 2 years. For the ± 10% sensitivity range, savings will vary between £773.79 (least favourable assumptions combined) and £981.45 (most favourable assumptions combined).

These savings estimates may easily be translated into national figures. Approximately 14 000 women die of breast cancer each year in England and Wales (Office of Population Censuses and Surveys, 1993), implying that, in the steady state, and assuming that all patients follow the above regimen, half of the 2 year savings will accrue to 28 000 cases each year. Thus, the estimated average savings at a national level would accordingly lie in the range £10.83–£13.74 million per annum. With perhaps more realism, however, a recent study of clinical practice (Gregory *et al.*, 1993) has suggested that only some 50% of patients with metastatic breast cancer actually receive *any* chemotherapy. Reworking the above calculation under the assumption that the economies in chemotherapy are only available for half the patients, we estimate that annual cost savings at a national level would lie in the range £8.16–£10.35 million.

### Discussion

Large though the projected savings are, it must be appreciated that they remain underestimates even under the model's most pessimistic assumptions. The data refer only to the most basic financial costs of assessment and therapy and, among salient omissions, are the following. First, the estimated costs of the chemotherapy regimens include those of patient preparation but *exclude* hospital overheads. Second, the assessment protocols are costed only on the basis of the tests administered and *exclude* both the input of the supervising physician (which is likely to be more considerable in the UICC case) and the cost of a radiologist experienced in assessing therapeutic response. Third, a reduction in the number of courses of chemotherapy would also economise on the number of routine investigations necessary to assess patients before each course. Finally, savings on in-patient costs as a result of earlier discontinuation of treatment whenever appropriate have not been included, and studies conducted in other hospitals suggest that such costs are a major determinant of the overall cost of treatment (de Koning *et al.*, 1992; Hurley *et al.*, 1992; Richards *et al.*, 1993).

This having been said, it is important to stress that the model-building approach adopted above cannot, in the final analysis, hope to supplant important evidence which would only be obtainable in a future trial context. Among the 'unknowns' at this stage are the following. First, we have presumed that our interpretation of the UICC criteria is the appropriate definition of current clinical practice. Whether the full range of investigations encompassed by the UICC criteria is, in fact, clinically the most appropriate is beyond the scope of this paper, although it is evident that, at institutions where a more restricted interpretation of the criteria is employed, the potential cost savings of serum marker monitoring would be smaller. Second, there could exist circumstances under which these calculations *overstate* the true economies of serum marker monitoring. For example, closer monitoring might lead, in practice, to an earlier switch from (generally cheap) hormone therapy to (generally more expensive) chemotherapy, although such cost increases might eventually be offset by improved quality of life.

Related to this point is the fact that the model has not been extended to include the cost implications of third- or fourth-line chemotherapy regimens, because their use in Nottingham is largely confined to the palliation of symptoms in only a few, quite specific cases. Despite limited evidence of efficacy, other hospitals might well make routine use of such regimens, which are generally more expensive than first- and second-line therapy. However, the model suggests that corresponding savings should be available for third- and fourth-line chemotherapy, in cases where it is employed more regularly, and, overall, this would tend to increase the economies available to the serum markers regimen.

Third, our cost simulation has been based on an assumed survival time of 2 years. Patients surviving longer than this median will naturally offer scope for greater cost economies, while earlier deaths would result in smaller cost savings. The final net effect will be determined by the experimentally observed survival distribution and, other things being equal, a skew to the right would entail smaller potential savings.

Fourth, it is conceivable that, in particular cases, additional investigations would be required for the purposes of monitoring therapy, over and above those suggested by our models of the UICC and serum marker criteria. These additional potential costs cannot be accommodated in the model, in the absence of experimental data, although, unless the use of further investigations is disproportionate between criteria, there seems no reason to believe that such costs would affect the scale of estimated cost savings. Finally, it is possible that different hospitals would make use of drugs different from those typically employed in Nottingham and cost savings would be smaller were cheaper drugs to be employed in chemotherapy. For example, the use of doxorubicin treatments, at £221.73 per month, in place of MMM and epirubicin, would reduce the first- and second-line chemotherapy savings in the Table II model by around 11% and 21% respectively. On the other hand, other centres might currently be employing more expensive drug regimens than Nottingham, in which case the savings from serum markers would be greater.

It must again be stressed that our estimates of cost savings are valid only within the confines of the model's assumptions and the data collected at Nottingham. It accordingly remains to be demonstrated more rigorously that the outcomes of serum marker assessment are at least as reliable as those of UICC and that our cost estimates are applicable to hospitals in general. Such a demonstration would only be possible as a result of a multicentre, randomised controlled trial, comparing two groups of patients treated on the basis of either UICC or tumour marker assessment. The magnitude of the projected cost savings certainly supports the case for such a trial; indeed, based on our estimates, even a large, multicentre trial could pay for itself in a matter of weeks.

### References

COOPER EH, FORBES MA, HANCOCK AK, PRICE JJ AND PARKER D. (1989). The evolution of mucin-like carcinoma associated antigen (MCA) in breast cancer. *Br. J. Cancer*, **59**, 797-800.

DALY L, FERGUSON J, CRAM GP, HARS V, GEORGE SL, McCARTY KS AND BAST RC. (1992). Comparison of a novel assay for breast cancer mucin to CA15-3 and carcinoembryonic antigen. *J. Clin. Oncol.*, **10**, 1057-1065.

DE KONING HV, VAN INVELD BM, DE HAES JCJM, VAN OORTMARSSEN GJ, KLIJN JGM AND VAN DER MAAS PJ. (1992). Advanced breast cancer and its prevention by screening. *Br. J. Cancer*, **65**, 950-955.

DIXON AR, JONROP I, JACKSON L, CHAN SY, BADLEY RA AND BLAMEY RW. (1991). Seriological monitoring of advanced breast cancer treated by systemic cytotoxics by a combination of CEA, CA15-3 and ESR: fact or fiction? *Dis. Markers*, **9**, 167-174.

DNISTRIAN AM, SCHWARTZ MK, GREENBERG EJ, SMITH CA AND SCHWARTZ DC. (1991). CA15-3 and carcinoembryonic antigen in the clinical evaluation of breast cancer. *Clin. Chim. Acta*, **200**, 81-93.

GREGORY WM, SMITH P, RICHARDS MA, TWELVES RK AND RUBENS RD. (1993). Chemotherapy of advanced breast cancer: outcome and prognostic factors. *Br. J. Cancer*, **68**, 988-995.

HAYWARD JL, CARBONE PP, HEUSON JC, KUMAOKA S, SEGALOFF A AND REUBENS RD. (1977). Assessment of response to therapy in advanced breast cancer: a project of the programme on clinical oncology of the International Union Against Cancer, Geneva, Switzerland. *Cancer*, **39**, 1289-1294.

HURLEY SF, HUGGINS RM, SNYDER RD AND BISHOP JF. (1992). The cost of breast cancer recurrences. *Br. J. Cancer*, **65**, 449-455.

LAURENCE V, FORBES MA AND COOPER EH. (1991). Use of mucin-like cancer associated antigen (MCA) in the management of breast cancer. *Br. J. Cancer*, **63**, 1000-1004.

OFFICE OF POPULATION CENSUSES AND SURVEYS. (1993). *Mortality Statistics: Causes*. Series DH2, No. 18. London: HMSO.

RICHARDS MA, BRAYSHER S, GREGORY WM AND RUBENS RD. (1993). Advanced breast cancer: use of resources and cost implications. *Br. J. Cancer*, **67**, 856-860.

ROBERTSON JRF, WILLIAMS MR, TODD JH, NICHOLSON RI, MORGAN DAL AND BLAMEY RW. (1989). Factors predicting the response of patients with advanced breast cancer to endocrine (Megace) therapy. *Eur. J. Cancer Clin. Oncol.*, **25**, 469-475.

ROBERTSON JRF, PEARSON D, PRICE MR, SELBY C, BADLEY RA, PEARSON J, BLAMEY RW AND HOWELL A. (1990). Assessment of four monoclonal antibodies as serum markers in breast cancer. *Eur. J. Cancer*, **26**, 1127-1132.

ROBERTSON JRF, PEARSON D, PRICE MR, SELBY C, PEARSON J, BLAMEY RW AND HOWELL A. (1991*a*). Prospective assessment of the role of five tumour markers in breast cancer. *Cancer Immunol. Immunother.*, **33**, 403-410.

ROBERTSON JRF, PEARSON D, PRICE MR, SELBY C, BLAMEY RW AND HOWELL A. (1991*b*). Objective measurement of therapeutic response in breast cancer using tumour markers. *Br. J. Cancer*, **64**, 757-763.

TONDINI C, HAYES DF, GELMAN R, HENDERSON IC AND HUFE DW. (1988). Comparison of CA15-3 and carcinoembryonic antigen in monitoring the clinical course of patients with metastatic breast cancer. *Cancer Res.*, **48**, 4107-4112.

WILLIAMS MR, TURKES A, PEARSON D, GRIFFITHS K AND BLAMEY RW. (1990). An objective biochemical assessment of therapeutic response in metastatic breast cancer: a study with external review of clinical data. *Br. J. Cancer*, **61**, 126.